# The shared circulating diagnostic biomarkers and molecular mechanisms of systemic lupus erythematosus and inflammatory bowel disease

**DOI:** 10.3389/fimmu.2024.1354348

**Published:** 2024-05-07

**Authors:** Hao-Wen Sun, Xin Zhang, Cong-Cong Shen

**Affiliations:** ^1^ Department of Gastroenterology, Affiliated Hospital of Nantong University, Medical School of Nantong University, Nantong, China; ^2^ Department of Dermatology, Affiliated Hospital of Nantong University, Medical School of Nantong University, Nantong, China

**Keywords:** bioinformatic analysis, hub genes, immune cells infiltration, inflammatory bowel disease, systemic lupus erythematosus

## Abstract

**Background:**

Systemic lupus erythematosus (SLE) is a multi-organ chronic autoimmune disease. Inflammatory bowel disease (IBD) is a common chronic inflammatory disease of the gastrointestinal tract. Previous studies have shown that SLE and IBD share common pathogenic pathways and genetic susceptibility, but the specific pathogenic mechanisms remain unclear.

**Methods:**

The datasets of SLE and IBD were downloaded from the Gene Expression Omnibus (GEO). Differentially expressed genes (DEGs) were identified using the Limma package. Weighted gene coexpression network analysis (WGCNA) was used to determine co-expression modules related to SLE and IBD. Pathway enrichment was performed using Gene Ontology (GO) and Kyoto Encyclopedia of Genes and Genomes (KEGG) analysis for co-driver genes. Using the Least AbsoluteShrinkage and Selection Operator (Lasso) regressionand Support Vector Machine-Recursive Feature Elimination (SVM-RFE), common diagnostic markers for both diseases were further evaluated. Then, we utilizedthe CIBERSORT method to assess the abundance of immune cell infiltration. Finally,we used the single-cell analysis to obtain the location of common diagnostic markers.

**Results:**

71 common driver genes were identified in the SLE and IBD cohorts based on the DEGs and module genes. KEGG and GO enrichment results showed that these genes were closely associated with positive regulation of programmed cell death and inflammatory responses. By using LASSO regression and SVM, five hub genes (KLRF1, GZMK, KLRB1, CD40LG, and IL-7R) were ultimately determined as common diagnostic markers for SLE and IBD. ROC curve analysis also showed good diagnostic performance. The outcomes of immune cell infiltration demonstrated that SLE and IBD shared almost identical immune infiltration patterns. Furthermore, the majority of the hub genes were commonly expressed in NK cells by single-cell analysis.

**Conclusion:**

This study demonstrates that SLE and IBD share common diagnostic markers and pathogenic pathways. In addition, SLE and IBD show similar immune cellinfiltration microenvironments which provides newperspectives for future treatment.

## 1 Background

Inflammatory bowel disease (IBD) is a chronic, recurrent inflammatory disease of the gastrointestinal tract, which mainly includes ulcerative colitis (UC) and Crohn’s disease (CD). The most prevalent clinical manifestations of IBD include bloody stool, diarrhea, and recurrent stomach pain (1). At the same time, parenteral comorbidities like uveitis, arthritis, erythema nodosum, and systemic lupus erythematosus (SLE) may occur in patients with IBD (2, 3). Currently, IBD is mainly considered to be an idiopathic disease caused by a combination of genetic susceptibility, dysbiosis of the intestinal flora, and abnormal mucosal immune response (4), but the exact pathogenesis is still unclear.

SLE is an autoimmune disease that involves multiple organs throughout the body, is prevalent in women of childbearing age, and can lead to death and disability (5). Patients with SLE most commonly die from infections, lupus nephritis, lupus encephalopathy, and cardiovascular events (6). The pathogenesis of SLE is complex and consists of genetic and environmental factors, activation of cytokines and complement, and deposition of circulating immune complexes (7, 8). Their interaction ultimately leads to inflammation and systemic multi-organ damage. SLE may manifest independently or in conjunction with IBD (9, 10). In 1956, Brown et al. reported the first case of SLE in combination with UC (11). Previous studies indicated that the overall prevalence of UC was 0.4% in the SLE population, which is higher than in the general population (12). In a multicenter study conducted in Israel, 5018 patients with SLE were matched with 25090 normal people, and it was discovered that the SLE population had double the prevalence of CD as the control group (9). This suggests that SLE and IBD may share a common pathogenesis. Although autoimmune diseases are recognized as distinct entities, Noel Rose’s hypothesis of “common threads” indicates that there is a broad overlap in the pathophysiology and therapeutic strategies of autoimmune disorders (13). Patients with both SLE and IBD have been shown to have positive anti-neutrophil cytoplasmic antibodies, anti-lymphocytotoxic antibodies, anti-nuclear antibodies, and anti-ds-DNA antibodies (10, 14–16). It has also been demonstrated the positivity rate of anti-nuclear antibodies and anti-ds-DNA antibodies is as high as 100% in patients with both disorders, whereas the latter rate is only 49% in patients with SLE alone (10). In addition, HLA status is highly correlated with these two autoimmune diseases with a hereditary basis (17, 18). It is challenging to make a clinical diagnosis of IBD complicated with idiopathic SLE because patients with SLE or IBD have similar clinical manifestations and laboratory results (9). Meanwhile, medications used to treat IBD, such as sulfasalazine and infliximab, cause drug-induced lupus, and SLE patients with lupus vasculitis have IBD-like gastrointestinal reactions such as abdominal pain and diarrhea (10). Therefore, the identification of new diagnostic markers and therapeutic targets for the disease is crucial.

In recent years, with the development of bioinformatics technology, we can comprehensively analyze the potential relationship between SLE and IBD. In this paper, based on the sequencing data of the two diseases in the database, we used WGCNA to confirm the co-expression modules between IBD and SLE. Then we screened out candidate common driver genes of the two diseases and analyzed them by GO and KEGG to explore common biological pathways. Hub genes were identified using LASSO regression analysis and SVM-RFE and their predictive value was assessed. Furthermore, the hub gene expression profiles in various immune cells in the SLE population were investigated by using single-cell analysis.

## 2 Methods

### 2.1 Bulk transcriptome data preprocessing

According to the selection strategy based on previous literature (19–22), we ultimately identified four relatively large transcriptome datasets originating from peripheral blood samples: GSE72326, GSE81622, GSE3365, and GSE126124. For SLE, we included 157 SLE samples and 20 healthy control samples from GSE72326, as well as 15 SLE samples and 25 healthy control samples from GSE81622. For IBD, we included 85 IBD samples and 42 healthy control samples from GSE3365, along with 57 IBD samples and 32 healthy control samples from GSE126124. The datasets utilized in our research consisted of peripheral blood mononuclear cells (PBMCs). For the transcriptome data mentioned above, we conducted GeneSymbol mapping according to their respective platforms. In cases of multiple matches, we took the median value. The final expression matrix was obtained by normalizing using the log2(X+1) method. During preprocessing, after initial quality control checks, the ‘normalizeBetweenArrays’ function from the ‘limma’ package was utilized to perform quantile normalization. This method adjusts the expression values so that each sample has the same empirical distribution of expression values, effectively minimizing technical variation among samples. Subsequently, we included 11,350 genes that overlapped across the four datasets for further bioinformatics analysis (**Supplementary Figure S1**).

### 2.2 Single-cell transcriptome data

Due to the absence of PBMC sequencing data in IBD samples, we downloaded only the adult SLE dataset from GSE135779, which includes PBMC data from 7 adult SLE patients and PBMC data from 5 healthy adults as controls. The preprocessing of single-cell transcriptome data followed the methodology described in previous literature using the Seurat package (23). In brief, quality control was performed by filtering cells with nFeature_RNA > 200, nFeature_RNA < 2500, and percent.MT < 5. After normalization, analysis was conducted on the top 2000 highly variable genes in each sample following variance-stabilizing transformation. Data integration was carried out using the IntegrateData function, scaling with the ScaleData function, and dimension reduction with the RunPCA function. Finally, cell clustering was performed using the FindNeighbors and FindClusters functions. The AverageExpression function was used to obtain mRNA expression at the pseudo-bulk level for different samples and t-tests were performed to compare the differences in expression of key genes.

### 2.3 Preselection of diagnostic biomarkers

Differential gene expression (DEGs) analysis was conducted in the GSE72326 and GSE3365 datasets using the limma package (limma powers differential expression analyses for RNA-sequencing and microarray studies), with the cutoff criteria of P.adj.value < 0.05 and |LogFC| > 0.5. We utilized the Benjamini-Hochberg procedure to adjust the p-values for multiple testing. In the WGCNA analysis (24), all genes from the GSE72326 and GSE3365 datasets were used to construct an input matrix. Topological calculations were performed with soft thresholding ranging from 1 to 20, and the optimal soft threshold was determined. The relationship matrix was transformed into an adjacency matrix, then further transformed into a topological overlap matrix (TOM). Average linkage hierarchical clustering was applied to classify modules based on TOM, with each module containing no fewer than 100 genes. Similar modules were subsequently merged. Finally, Pearson’s method was used to calculate the correlation between the merged modules and disease occurrence, and the modules with the strongest positive and negative correlations with the disease were selected as core modules. Moreover, we defined gene significance (GS) as a measure of the association of individual genes with the trait of interest, and module membership (MM) as the measure of the correlation of gene expression profiles with the principal component of a given module.

### 2.4 GO and KEGG enrichment analysis

GO (Gene Ontology) and KEGG (Kyoto Encyclopedia of Genes and Genomes) enrichment analyses of common driver genes were performed using the clusterProfiler package (an R package for comparing biological themes among gene clusters). GO is used to annotate the biological processes, molecular functions, and cellular components of genes. Gene pathways were annotated using the KEGG. Enrichment was statistically significant when P < 0.05.

### 2.5 Construction of PPI networks

71 candidate common driver genes were entered into the String platform (https://string-db.org/) and independent genes were removed. Cytoscape was used to screen key genes and make a visual network. The above genes were used in Cytoscape software to calculate the TOP10 genes within the PPI network using the MCC algorithm (Identifying hub objects and sub-networks from complex interactome).

### 2.6 Machine learning selection of diagnostic biomarkers

Support Vector Machine (SVM) (25) and LASSO (26) were utilized as machine learning methods to identify core genes by removing feature vectors generated by the SVM. LASSO is a shrinkage estimator that refines the model by constructing a penalty function, compressing some regression coefficients, and handling biased estimates in the presence of multicollinearity. Seventy-one intersecting genes, after deduplication, underwent PPI network analysis. The top ten genes based on MCC were used as input for the expression profile. Disease occurrence was used as the classification variable, and SVM and LASSO were applied for biomarker selection.

### 2.7 Immune infiltration analysis

The CIBERSORT algorithm (27) was used to calculate the proportions of different immune cell types based on the expression levels of immune cell-related genes. The outputs of 22 infiltrating immune cell types were integrated to generate an immune cell composition matrix for analysis. Additionally, the Spearman method was employed to analyze the correlation between core biomarkers and the expression levels of infiltrating immune cells. The p-values have also been adjusted using the same Benjamini-Hochberg method.

### 2.8 Identification of drug candidates

The common hub genes of SLE and IBD were entered into the Enrichr platform (https://maayanlab.cloud/Enrichr/) (28). Then, we screen the drug candidates related to hub genes using Drug Signatures Database (DSigDB) (29).

## 3 Results

### 3.1 Identification of differentially expressed genes in SLE and IBD

**Figure 1:**
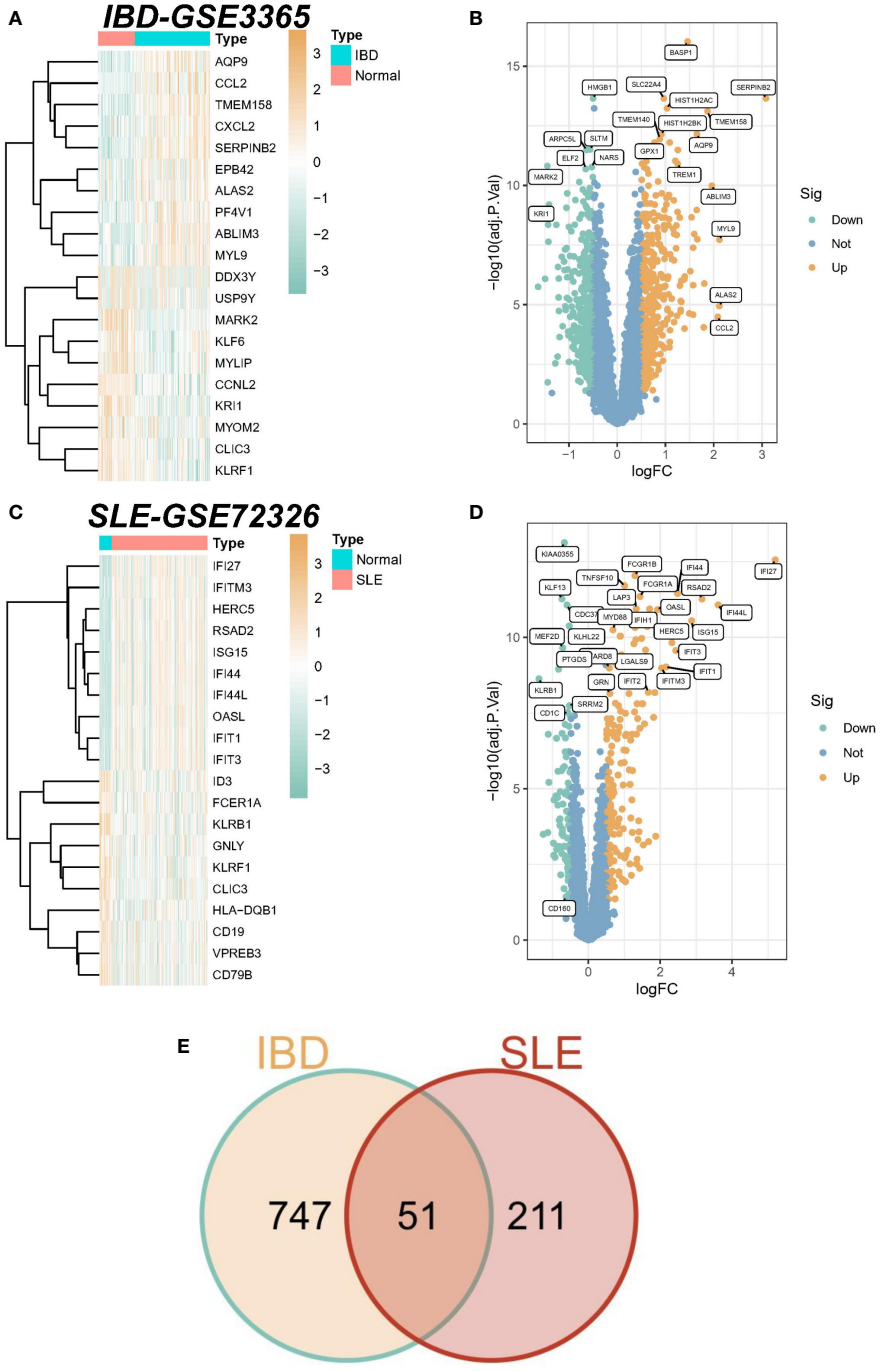
Identification of differentially expressed genes (DEGs). **(A)** Heatmap of the top 20 genes with the most prominent differential expression in the IBD-GSE3365 cohort. **(B)** Volcano plot showing the distribution of DEGs in IBD. Green color represents down-regulated genes and yellow color represents up-regulated genes. **(C)** Heatmap of the top 20 genes with the most pronounced expression difference in the SLE-GSE72326 cohort. **(D)** Volcano plot showing the distribution of DEGs in SLE. Green color represents down-regulated genes and yellow color represents up-regulated genes. **(E)** Venn diagram of overlapping DEGs in SLE versus IBD.

A total of 798 differential genes were identified based on the IBD dataset (GSE3365), and the heatmap demonstrated the top 20 genes with the most significant up-regulation and down-regulation (**Figure 1A**), and the volcano plot showed the identified differential genes including 417 up-regulated and 381 down-regulated genes, among which SERPINB2 was the most significant up-regulation gene in the IBD samples. (**Figure 1B**). In addition, 262 differential genes were obtained from the SLE dataset (GSE72326), comprising 179 up-regulation genes and 83 down-regulation genes (**Figures 1C, D**). Finally, a total of 51 overlapping DEGs were established in the SLE and IBD datasets. The list of specific differential and common genes is shown in **Supplementary File 1**.

### 3.2 Weighted gene coexpression network analysis of SLE and IBD

**Figure 2:**
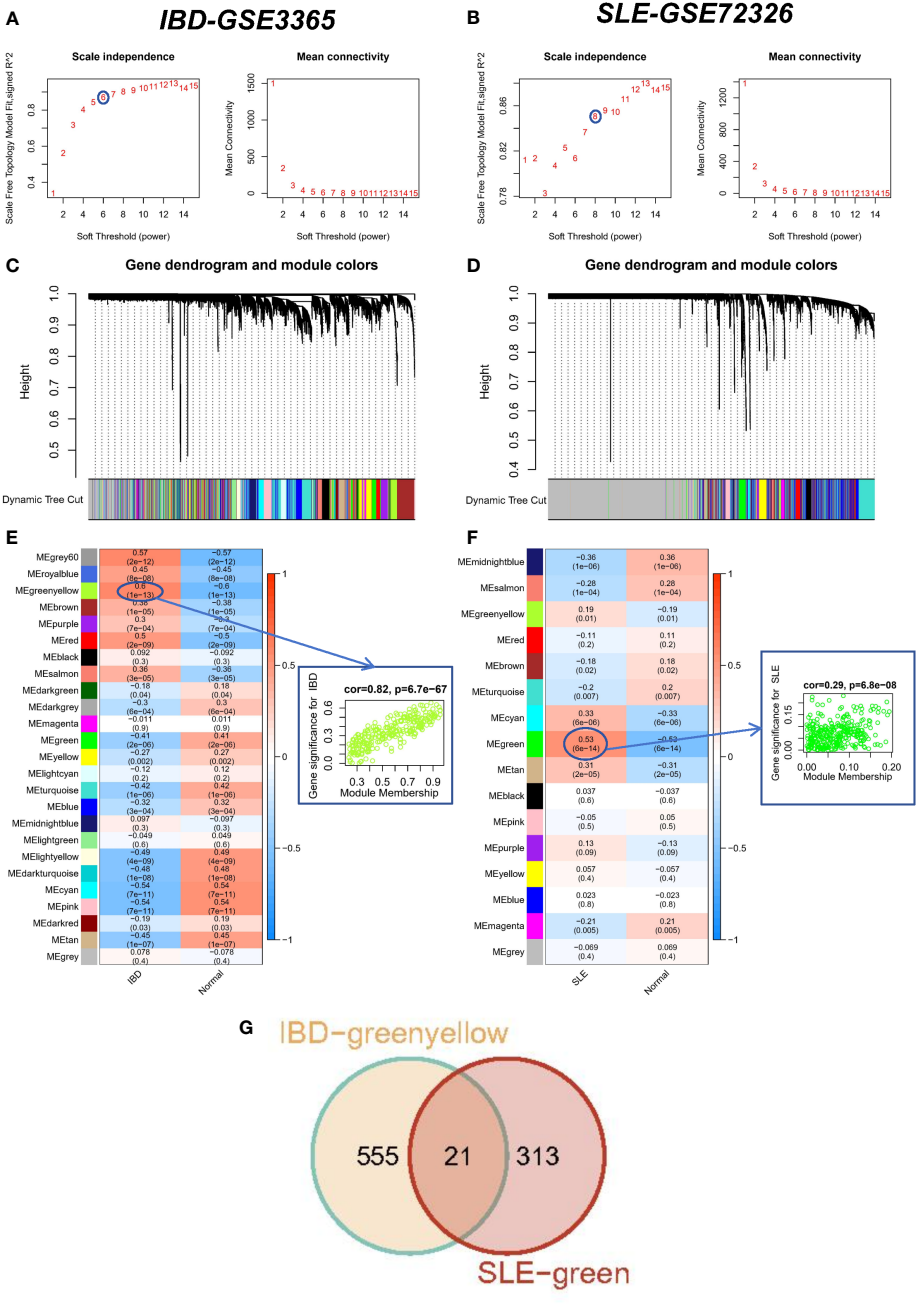
WCGNA analysis of SLE and IBD. **(A, B)** Mean connectivity for scale independence and soft threshold (β) in the IBD-GSE3365 cohort and the SLE-GSE72326 cohort. **(C, D)** Clustering dendrograms of genes in SLE and IBD. **(E, F)** Heatmap of the correlation analysis of module eigengenes with clinical phenotypes in SLE and IBD. Red color represents positive correlation and blue color represents negative correlation. **(G)** Venn diagram for intersecting genes between greenyellow module in IBD and green module in SLE.

We performed WGCNA on the IBD dataset GSE3365 and the SLE dataset GSE72326 to explore correlations between clinical traits and genes. There were no significant outlier samples in the SLE dataset and IBD dataset. According to the WGCNA method, the best soft threshold in the IBD dataset was 6, and the best soft threshold in the SLE dataset was 8 (**Figures 2A, B**). Based on the similarity between modules, 25 modules were finally determined in the IBD dataset as well as 16 modules were determined in the SLE dataset (**Figure 2C, D**). Then, the correlations between modules and traits were calculated, and we found that the greenyellow module had the strongest positive correlation with IBD (r=0.6) (**Figure 2E**), while the green module had the strongest positive correlation with SLE (r=0.53) (**Figure 2F**). More importantly, there was also a strong association between gene significance (GS) and module membership (MM) within the modules (the cor of IBD=0.82, the cor of SLE=0.29), reconfirming that the module genes significantly related to the occurrence of diseases. Ultimately, we discovered 21 overlapping genes by WGCNA that may drive the development of IBD and SLE (**Figure 2G**).

### 3.3 Enrichment analysis of SLE and IBD co-driver genes

**Figure 3:**
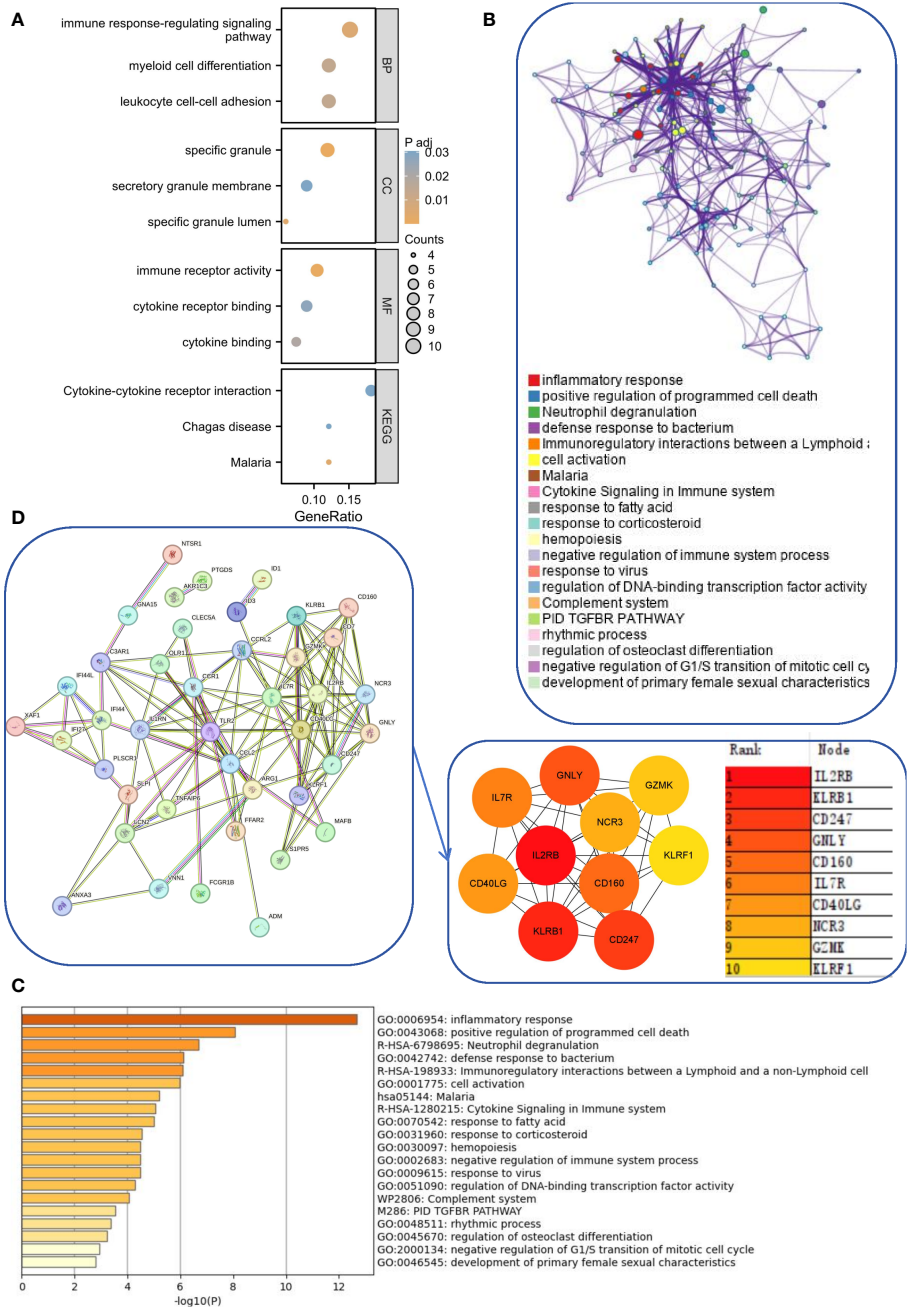
Functional enrichment and pathway enrichment of SLE and IBD co-driver genes. **(A)** GO analysis and KEGG analysis of common driver genes. **(B, C)** Enrichment analysis of 71 candidate common driver genes using Metascape online tool. **(D)** PPI network analysis of common driver genes.

There were 21 overlapping genes for the SLE and IBD modules and 51 shared genes for the DEGs. Considering that the modules screened from WGCNA contain a set of genes with analogous expression profiles, which may not cover the full range of DEGs or even be considerably distinct from the DEGs that may be crucial for disease progression, we integrated the DEGs with module genes to avoid omissions. After removing duplicates again, we acquired 71 candidate common driver genes (**Supplementary File 2**). These genes may play an important role in common molecular mechanisms involved in SLE and IBD. Therefore, we first performed GO and KEGG enrichment analyses for these genes. The results showed that these genes were substantially participating in cytokine-cytokine receptor interaction, immune response-regulating signaling pathway, myeloid cell differentiation and other pathways (**Figure 3A**). In addition, in order to further elucidate the enrichment pathways of the aforementioned peripheral circulation marker-related genes, we found that different genes in the metascape database may exhibit different functional group distributions, among which the positive regulation of programmed cell death and inflammatory responses is the most prominent (**Figure 3B**). At the same time, enrichment analysis based on the metascape database also showed a common role of immunity and inflammation in the etiology of IBD and SLE (**Figure 3C**). Finally, to further screen the genes into the same functional group, we entered 71 candidate common driver genes into the String database and deleted the independent genes. Subsequently, the MCC algorithm in Cytoscape software was used to determine the TOP 10 genes within the PPI network based on the aforementioned genes (**Figure 3D**). Finally, GNLY, IL7R, CD40LG, KLRB1, CD247, CD160, NCR3, GZMK, KLRF1, and IL2RB were determined as candidate diagnostic markers and IL2RB was the most significant within the group.

### 3.4 Identification and validation of potential shared hub genes by SVM and LASSO

**Figure 4:**
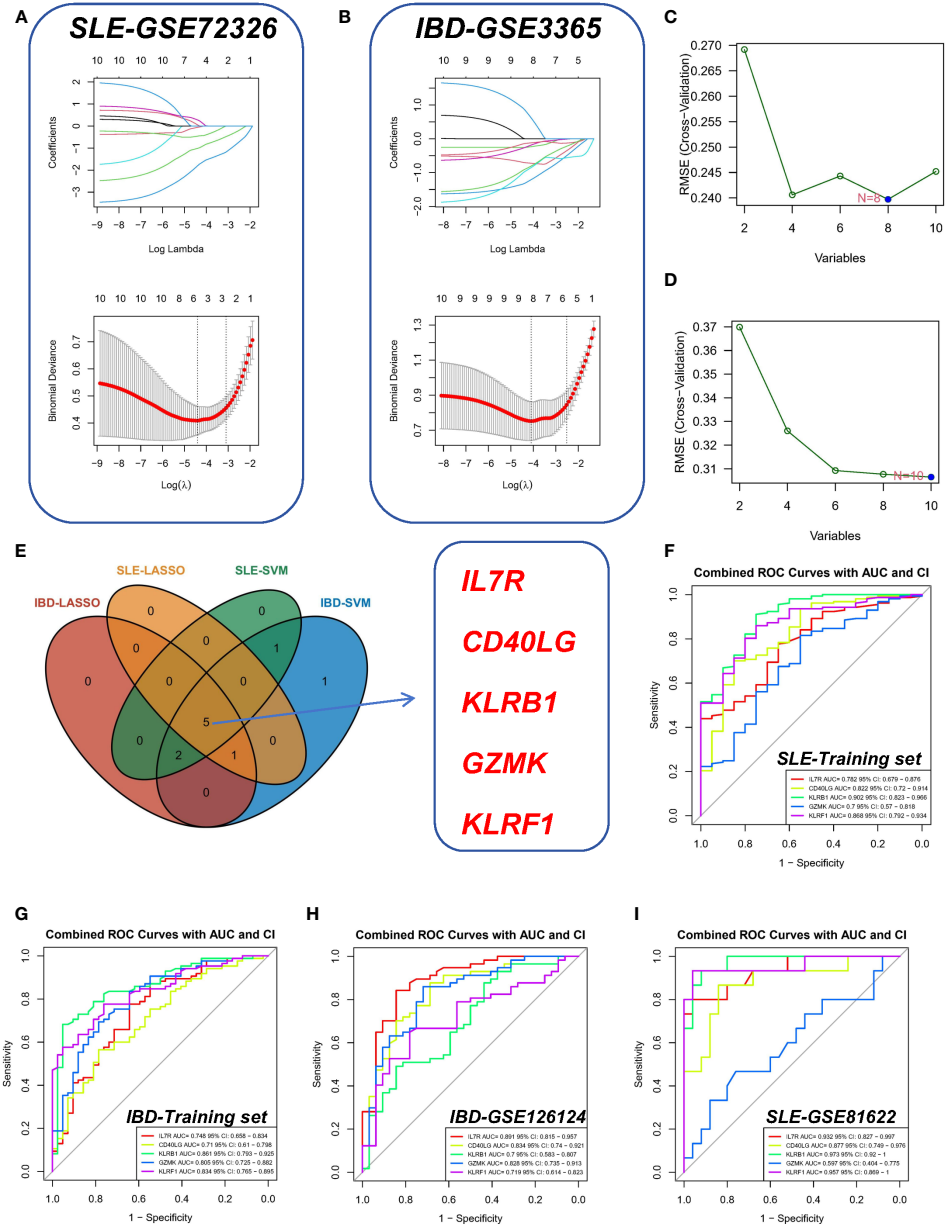
Identification of shared hub genes by SVM and LASSO. **(A, B)** LASSO regression analysis of the SLE-GSE72326 cohort and the IBD-GSE3365 cohort. **(C, D)** SVM analysis of the SLE and IBD cohorts. **(E)** Cross-identification of optimal shared hub genes using SVM and LASSO. **(F)** ROC curves for five shared diagnostic markers in the IBD-GSE3365 cohort. **(G)** ROC curves for five shared diagnostic markers in the SLE-GSE72326 cohort. **(H)** ROC curves for five shared diagnostic markers in the IBD-GSE126124 cohort. **(I)** ROC curves for five shared diagnostic markers in the SLE-GSE81622 cohort.

To further screen the pivotal genes with the most diagnostic value, we selected the most important features based on a machine learning algorithm. Among the above 10 candidate genes, SVM and LASSO regression analysis were performed successively. The LASSO approach was used to screen 6 genes in the SLE dataset (**Figure 4A**) and 8 genes in the IBD dataset (**Figure 4B**). Meanwhile, the SVM method was applied to filter out 8 genes from the SLE dataset (**Figure 4C**), whereas the IBD dataset preserved all 10 genes because irrelevant genes were not filtered out using SVM (**Figure 4D**). The genes filtered by the above different methods in different datasets were overlapped with each other, and five common diagnostic markers (KLRF1, GZMK, KLRB1, CD40LG, and IL7R) were finally identified (**Figure 4E**). Among them, IL7R, CD40LG, KLRB1, and GZMK were derived from common differential genes, while KLRF1 was derived from WGCNA.

Furthermore, we used ROC curves (**Figures 4F, G**) to assess the diagnostic predictive value of Hub genes in different datasets (**Figures 4F, G**). Of these, the AUC values of KLRF1 (AUC=0.868), GZMK (AUC=0.700), KLRB1 (AUC=0.902), CD40LG (AUC= 0.822), KLRB1 (AUC=0.902), CD40LG (AUC=0.782), and IL7R (AUC=0.782) in the SLE-GSE72326 dataset were all greater than 0.7 (**Figure 4F**). Similarly, the AUCs of KLRF1 (AUC=0.834), GZMK (AUC=0.805), KLRB1 (AUC=0.861), CD40LG (AUC=0.710), and IL7R (AUC = 0.748) in the IBD-GSE3365 dataset likewise showed their values more than 0.7 (**Figure 4G**). This indicates that these five genes have good diagnostic performance and may become common diagnostic markers for SLE and IBD.

**Figure 5:**
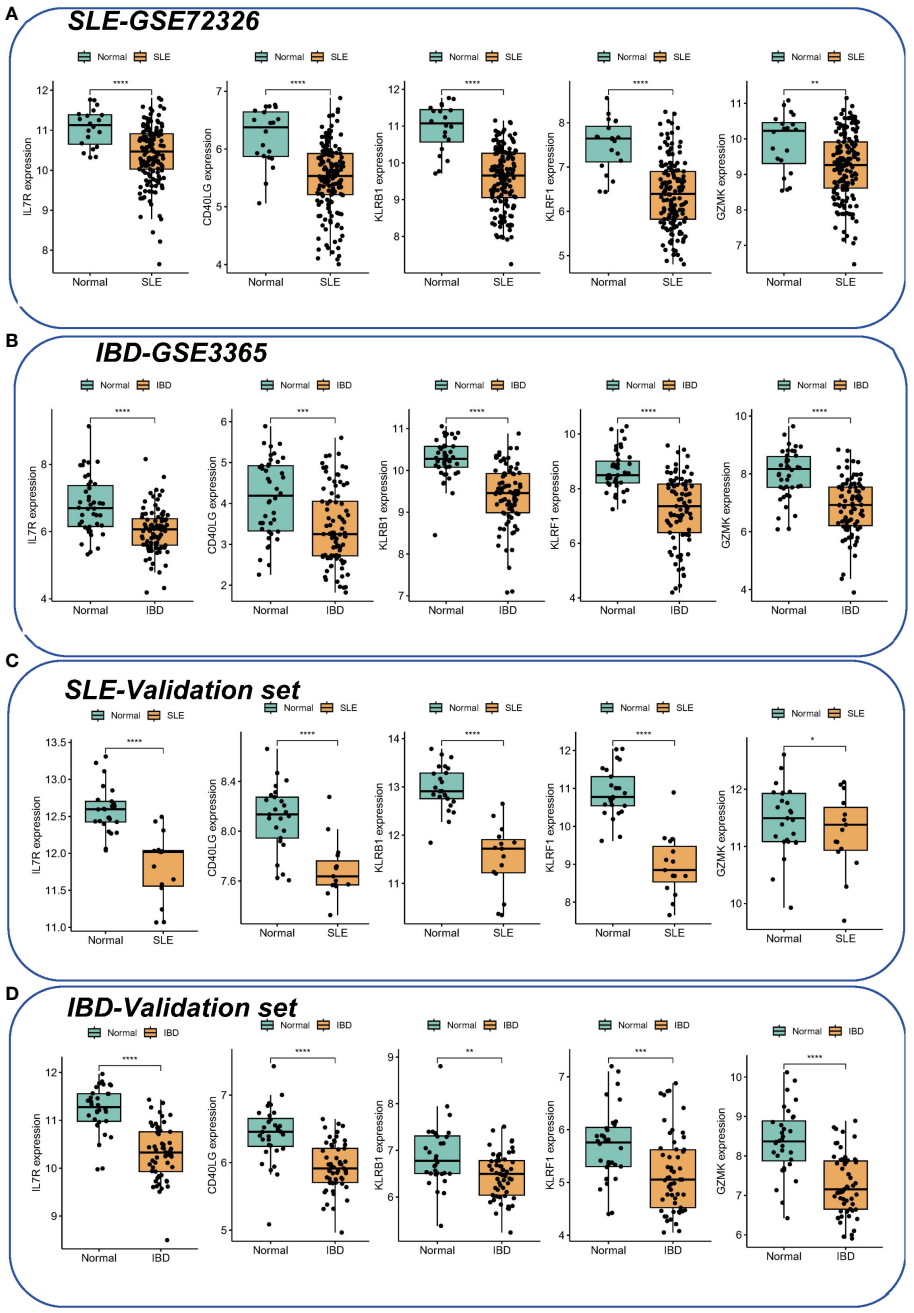
The expression of hub genes in SLE and IBD. **(A, B)** Expression of five hub genes in SLE-GSE72326 and IBD-GSE3365. **(C, D)** Expression of five hub genes in SLE-GSE81622 and IBD-GSE126124. Green color represents normal people and yellow color represents SLE/IBD patients. *p< 0.05; **p< 0.01; ***p< 0.001; ****p< 0.0001.

In the validation set, the AUCs of different cohorts also demonstrated good predictive efficacy (**Figures 4H, I**), in which the AUCs of KLRF1, GZMK, KLRB1, CD40LG, and IL7R were 0.719, 0.828, 0.700, 0.834, and 0.891, respectively, in the IBD validation set (GSE126124) (**Figure 4H**), and in the SLE validation set (GSE81622), the AUCs of all diagnostic markers were greater than 0.8, except for GZMK, whose AUC was less than 0.700 (**Figure 4I**). Subsequently, the boxplots showed that all five diagnostic markers in the SLE and IBD training set were significantly down-regulated in the disease group (**Figures 5A, B**). More importantly, a consistent trend of differences was shown in the SLE validation set as well as the IBD validation set (**Figures 5C, D**).

### 3.5 Identification of candidate drugs based on hub genes

Based on the DSigDB library in Enrichr, four drugs (choline/aspirin/ARSENIC/Mustard gas) with significant P-values after correction were screened by calculating P-values and the binding scores to core hub genes. These potential small molecule compounds could be applied as co-treatments for IBD and SLE (**Table 1**).

**Table 1:** Identification of candidate drugs for the treatment of SLE and IBD based on the hub genes.

Term	P-value	Adjusted P-value	Odds Ratio
choline CTD 00005662	6.34E-05	0.007676057	271.3741497
aspirin CTD 00005447	2.10E-04	0.012732687	52.25
ARSENIC CTD 00005442	7.25E-04	0.029230461	33.78529412
Mustard gas CTD 00006356	0.00111561	0.033747216	62.21069182

### 3.6 Immune cell infiltration and its correlation with shared hub genes

**Figure 6:**
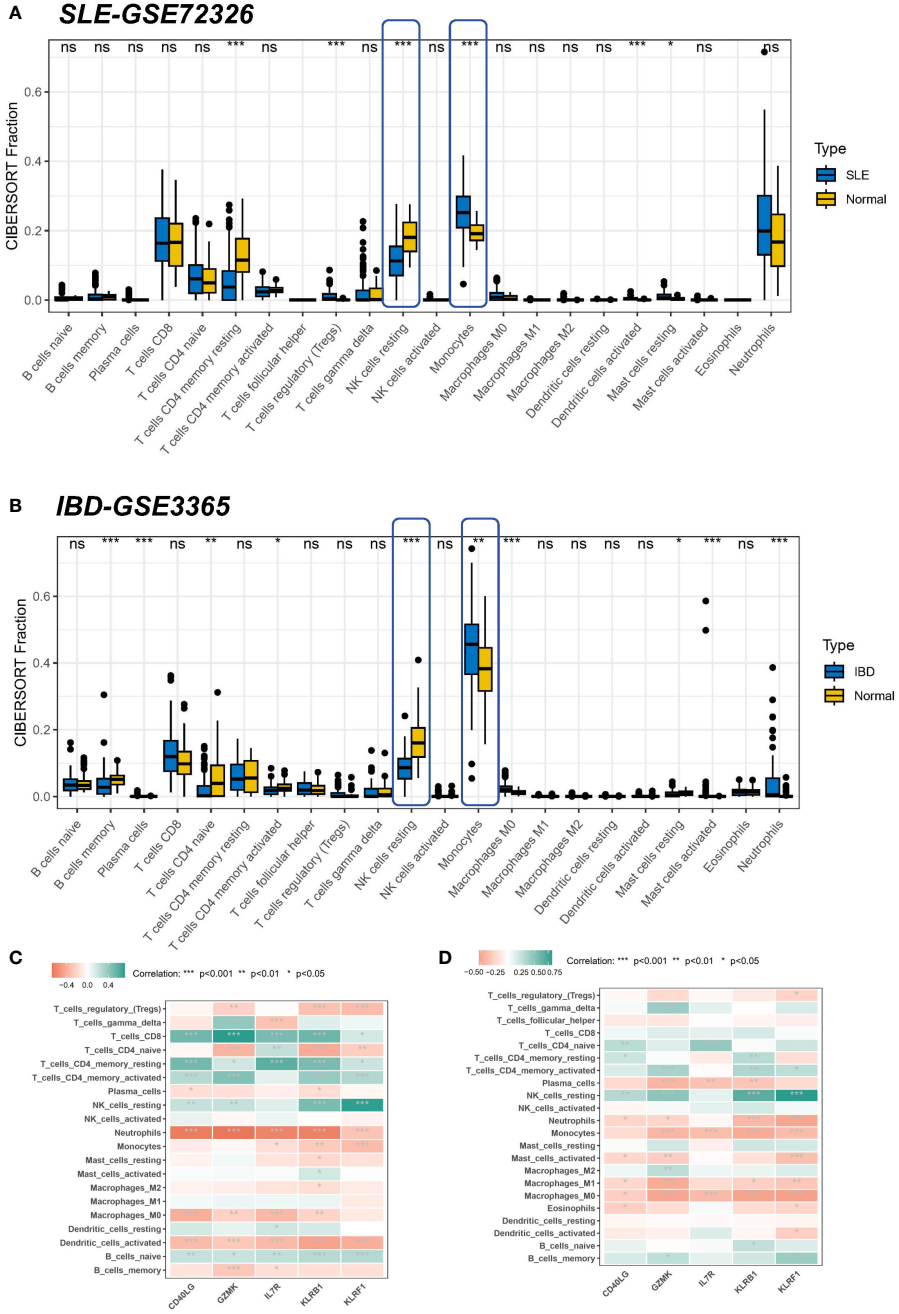
Correlation of hub genes and immune cell infiltration in SLE and IBD. **(A, B)** Boxplots showing the pattern of immune cell infiltration in the SLE-GSE72326 cohort and the IBD-GSE3365 cohort. Blue color represents SLE/IBD patients and yellow color represents normal people. **(C, D)** Heatmaps showing the correlation between hub genes and immune cells. Green color represents positive correlation and red color represents negative correlation. *p< 0.05; **p< 0.01; ***p< 0.001; ns, non-significant.

Since enrichment analyses demonstrated that immunity is vital for the development of both diseases, we investigated whether different patterns of immune infiltration could be recognized by the CIBERSORT method based on 22 types of immune cells. First, we evaluated the SLE dataset and the IBD dataset. Differential expression analysis showed that SLE and IBD showed consistent differential trends compared to normal samples. More precisely, compared to healthy human blood samples, monocytes were significantly higher in SLE and IBD, while resting NK cells were significantly downregulated (**Figures 6A, B**). Interestingly, this suggests that immune dysregulation and inflammatory responses seem to occur in both SLE and IBD.

However, common differences in the proportion of immune cell composition are only one aspect of the shared pathogenesis of SLE and IBD. We still need to confirm whether these five shared pivotal genes are associated with immune infiltration in the peripheral blood, and if so, which immune cells they are in particular associated with, as well as to determine their commonality. Correlation analysis, therefore, revealed that IL7R, KLRB1, and KLRF1 were negatively linked with neurophils in the SLE dataset (**Figure 6C**). Meanwhile, a similar result was seen in the IBD dataset, where GZMK, CD40LG, KLRB1, and KLRF1 were negatively related to neurophils, whereas the remaining four common markers aside from IL7R were positively linked with resting NK cells (**Figure 6D**). As most of the core genes are low-expressed in the disease group (SLE or IBD), it also means that there will be more neutrophils enriched in the disease. This again suggests that hub genes may be involved in regulating autoimmunity by modulating the expression of immune cells.

### 3.7 Single-cell analysis of hub gene locations

**Figure 7:**
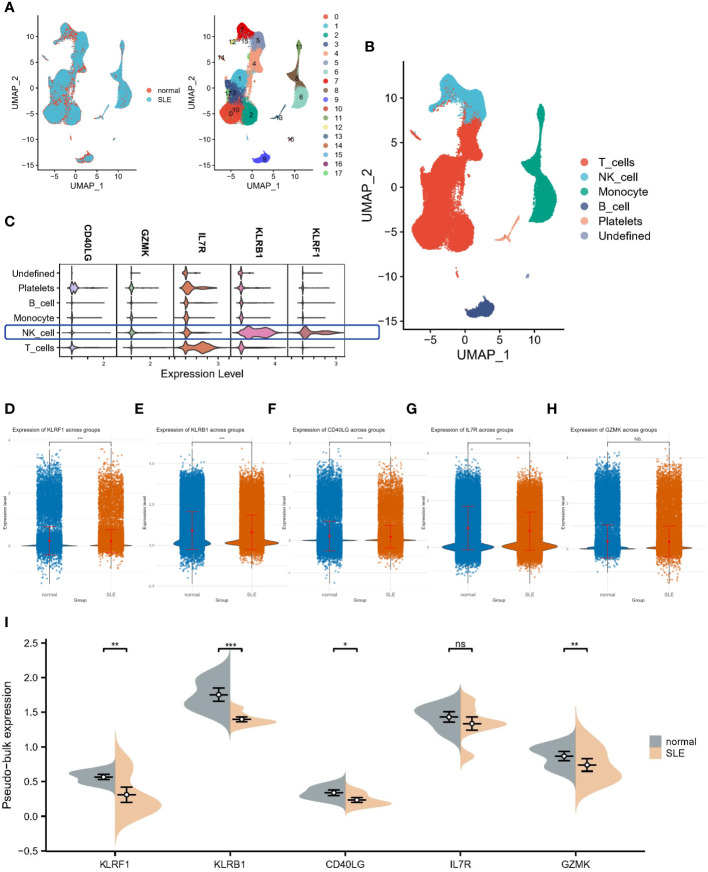
Single-cell analysis of hub gene expression profiles. **(A)** UMAP visualization of 18 circulating cells in SLE. **(B)** UMAP visualization of immune cells in SLE. **(C)** Localization analysis of hub genes. **(D–H)** Cellular expression distribution of the five hub genes in controls and SLE patients. **(I)** Differential analysis based on single-cell pseudo-bulk data. *:p< 0.05; **:p< 0.01; ***:p< 0.001; ns: non-significant.

All circulating cells were categorized into 18 clusters following quality control on the 12 samples in the SLE dataset (**Figure 7A**). As previously described in the literature, the signatures of CD3E, IL7R, CCR7, CD4, CD8A, and CCL5 were selected for T cell annotation; the signatures of KLRB1, NKG7, and GNLY were selected for NK cell annotation; the signatures of LYZ, CD14, CD68, S100A9, CD16, FCGR3A and CD1C were selected for monocyte annotation; and the signatures of MS4A1, CD19 and CD79A were selected for B-cell annotation. Ultimately, we identified six cell populations, which contained one undefined cell population (**Figure 7B**). Subsequently, we performed localization analysis of key diagnostic markers, and we found that the majority of the markers were commonly expressed in NK cells (**Figure 7C**). A reanalysis of the different samples revealed that, as illustrated by the Bulk transcriptome data, KLRF1 (**Figure 7D**), KLRB1 (**Figure 7E**), CD40LG (**Figure 7F**) and IL7R (**Figure 7G**) exhibited decreased expression in SLE. Whereas, IL7R expression was not significantly different among samples (**Figure 7H**). Moreover, pseudobulk comparison allows for a more appropriate comparison between patient groups by averaging the gene expression data, thereby minimizing the noise and variability inherent in single-cell data. Therefore, we again performed a differential analysis based on single-cell pseudo-bulk data to probe the expression of the five genes mentioned above. Consistently, all genes except IL7R were downregulated in expression in SLE samples (**Figure 7I**).

## 4 Discussion

SLE and IBD are both autoimmune diseases and can occur at the same time. Usually, SLE is diagnosed before IBD (10). It has been reported that persistent diarrhea caused by UC is associated with SLE (30), indicating that one autoimmune disease may be comorbid with other autoimmune diseases. Shared autoantibodies and HLA-associated genetic susceptibility may be a common pathogenic mechanism for SLE and IBD, but the latter remains controversial. In a study with 1305 SLE patients, mutations in the risk allele for IBD, CARD15, were found to have a strong effect on the risk of developing SLE (31), but the opposite results were obtained in another study (32). In addition, differential diagnosis of IBD combined with idiopathic SLE is challenging because of the intersecting clinical manifestations of the two diseases. patients with SLE often present with gastrointestinal symptoms such as abdominal pain, diarrhea, and black stools, which may be due to the patient’s concurrent combination with IBD; however, it is necessary to rule out SLE-caused conditions such as gastrointestinal vasculitis, intestinal peristalsis, plasma membrane inflammation, and pancreatitis (33). Musculoskeletal manifestations are also the most common extraintestinal complications of IBD, with peripheral arthritis and non-inflammatory arthralgia being the most common, and may be accompanied by generalized fibromyalgia and dactylitis (34), which further increases the difficulty of clinical differential diagnosis. Therefore, identification of shared biomarkers and pathogenesis is crucial for the diagnosis and treatment of the diseases.

Firstly, we identified 51 overlapping DEGs in the IBD and SLE datasets and used WCGNA to determine the most strongly associated modules. By integrating the WCGNA modules and DEGs, 71 candidate co-driver genes were finally screened. The enrichment results of GO and KEGG showed significant up-regulation of cytokine-cytokine receptor interactions, immune response modulation signaling pathways, and myeloid differentiation signaling pathways, suggesting that these genes are involved in the regulation of immune responses. Immune system dysregulation could be a major factor in the development of SLE and IBD. Subsequently, enrichment analysis of common driver genes based on the metascape database illustrated the same results, and these genes were most closely linked to positive regulation of programmed cell death (PCD) and inflammatory response.

PCD is the genetically controlled, autonomous and orderly death of cells in order to maintain the stability of the intracellular environment (35). Apoptosis, necroptosis, autophagy, pyroptosis, and necrosis are the main forms of cell death (36). Numerous studies have shown that SLE may be an autoimmune disease caused by cellular debris undergoing PCD (37). In SLE patients, there is a defect in apoptosis, which then leads to secondary cell necrosis (38, 39). The former is thought to be immunologically silent, but necrotic cellular debris is pro-inflammatory (40). Once cells are fragmented, increased release of nucleosomes will lead to autoantibody production as well as deposition of immune circulating complexes (ICs), thus triggering an autoimmune response (41). Miao et al. found that, besides dysregulated apoptosis and necrotic cell death, cellular pyroptosis also plays an important role in the pathogenic process of SLE. Neutrophils are activated in SLE patients, and their release of nuclear DNA and mitochondrial DNA (mtDNA) is increased. This leads to the oligomerization of the pore-forming protein gasdermin D (GSDMD) to form pore membranes, which releases extracellular DNA and inflammatory mediators as well as promotes the development of SLE (42). Besides, immune-inflammatory mechanisms are essential in the pathogenesis of IBD. In patients with IBD, T cells in the intestinal lamina propria are overactivated, releasing increased proinflammatory cytokines, such as TNF-α. This contributes to PCD of intestinal epithelial cells, disruption of the mucosal barrier, and inflammatory responses to intestine (43–45). Kaser et al. demonstrated that the endoplasmic reticulum stress-induced unfolded protein response (UPR) may be an additional reason for the onset and persistence of intestinal inflammation in IBD. Defects in the UPR-related gene XBP1 directly lead to the activation of key pro-inflammatory pathways in the intestine, making mice more susceptible to IBD (46).

Then, based on 71 common driver genes, we used Cytoscape software to construct a PPI network to identify Hub genes and finally selected 10 genes as candidate diagnostic markers. To further screen the most diagnostic Hub genes, we used SVM and LASSO regression analysis to identify the best diagnostic biomarkers. Among them, five genes, KLRB1, KLRF1, GZMK, IL-7R and CD40LG, showed good diagnostic performance which was confirmed by ROC curves in the training and validation sets of IBD and SLE cohorts, respectively. More significantly, all five genes showed a consistent down-regulation trend in both IBD and SLE patients compared to controls.

KLRB1 (killer cell lectin-like receptor B1) is a member of the KLR gene family and encodes a C-type lectin receptor (CD161) to recognize ligands (47). As an inhibitory immune receptor, KLRB1 is commonly expressed on CD4+, CD8+, NK cells, NKT cells, and other T-cell subsets (48). Almost all inhibitory immunoreceptors contain immunoreceptor tyrosine-based inhibitory motifs (ITIMs) in their cytoplasmic tails to dock downstream effectors that mediate cell proliferation, differentiation, and cytotoxicity (49). For example, KLRB1 can interact with the ligand Lectin-like transcript 1 (LLT1) to inhibit NK cell toxicity (48, 50). Previous studies have demonstrated that KLRB1 expression is downregulated in SLE (51–53), which is also consistent with the results of this paper. Apart from SLE, aberrant KLRB1 expression has been correlated with other autoimmune diseases and inflammatory responses, including rheumatoid arthritis (RA) (54, 55), multiple sclerosis (MS) (56), and sepsis (57). Nevertheless, the expression of KLRB1 is not entirely consistent in these immunoinflammatory diseases. KLRB1 expression is up-regulated in early RA (54) and MS (56) and down-regulated in advanced RA (55) and sepsis (57). The exact mechanism remains unclear, but KLRB1 may develop into a biological marker specific to autoimmune diseases in the future. KLRF1 (killer cell lectin-like receptor F1) is an activated homodimeric C-type lectin-like receptor (CTLR) and a member of the KLR gene family (58). It is expressed in the vast majority of NK cells and is identified as a marker of mature NK cells in secondary lymphoid tissue (59). KLRF1 suppresses NK cells and monocytes by interacting with MHC class I ligands to influence autoimmunity (60). A previous study showed that KLRF1 was able to inhibit the secretion of inflammatory mediators such as TNF and IFN-γ (61). Perhaps KLRF1 could serve as a potential therapeutic target for IBD and SLE in the future. GZMK (Granzyme K) is a granule-secreting enzyme of the serine protease family that induces cell death and regulates the inflammatory response (54, 62). Although GZMK induces the secretion of several pro-inflammatory factors, including IL-1β, IL-6, IL-8 and TNF-α, and secretes proteases that degrade the extracellular matrix and amplify the inflammatory response (63–67), GZMK also reduces mature dendritic cells and antigen presentation to inhibit the body’s immune response (68), which may be an important pathogenetic mechanism of SLE and IBD caused by downregulation of GZMK. IL7R (interleukin 7 receptor) is a heterodimeric complex composed of IL-7Rα and γ chains (69) and is involved in autoimmune diseases such as IBD (70, 71), RA (72), and MS (73). It is also a risk factor for the development of hyperimmune and inflammatory responses in the body (69). It has been shown that IL7R plays a role in the pathogenesis of IBD, whose polymorphisms affect the risk of UC (71, 74, 75). Vranova et al. found that the IL-7/IL-7R pathway regulates tissue fluid homeostasis, body inflammation and autoimmunity. On the one hand, IL-7/IL-7R promotes the proliferation of memory T cells and enhances the cytotoxicity of CD8+ T cells, inducing excessive immune responses. On the other hand, IL-7/IL-7R stimulates lymphangiogenesis, promotes lymphatic return, and attenuates inflammatory responses by removing excess tissue fluid and inflammatory mediators in mice (76). Perhaps, activation of the IL-7R signaling pathway to reduce tissue inflammation could be an emerging therapeutic strategy for SLE and IBD. CD40LG, also known as CD40L, is a type II transmembrane protein that is crucial for regulating autoimmunity and cell death (77, 78). CD40L is subject to various types of genetic mutations and is highly heterogeneous (79). In a prior case report, the researcher found that downregulation of CD40L caused by missense mutations might induce various autoimmune diseases including IBD (80). Taken together, the five hub genes (KLRB1, KLRF1, GZMK, IL7R and CD40LG) all participate in the regulation of autoimmune and inflammatory responses and may be potential biomarkers for SLE with IBD.

Taking into account the important role of immune cells in the pathogenesis of SLE and IBD, we analyzed the patterns of immune cell infiltration in both diseases using CIBERSORT. The results showed that SLE and IBD present a similar immune infiltration landscape. More specifically, there was a significant increase in monocytes and a significant decrease in resting NK cells in SLE versus IBD. Previous studies have shown that patients with SLE have a decreased total number of NK cells and suppressed cytotoxicity, which may be correlated with IFN-α-mediated cell death (81–84). The decrease of NK cells is an important risk factor for the development of lupus nephritis and disease activity (85, 86). Similarly, Bittencourt et al. discovered that NK cells in IBD patients were functionally impaired, had diminished cytotoxicity, and expressed different killer cell immunoglobulin-like receptors (KIRs). Dysfunctional NK cells can release more pro-inflammatory cytokines including IL-17A and TNF-α, exacerbating the inflammatory response in patients (87). Monocytes can differentiate into macrophages and dendritic cells (DCs) in the periphery. These innate immune cells play an important role in the pathogenesis of SLE and IBD. Recently, it has been shown that plasmacytoid dendritic cells (pDCs) from SLE patients produce large amounts of IFN-α, which, upon binding to the receptor, activates the JAK-STAT signaling pathway and positively feedback stimulates the activation of pDCs and T cells (88). Moreover, an imbalance of macrophage polarization and aberrant activation underlie the development of SLE (89, 90). Recently, Hegarty et al. put forward the idea that monocytes and macrophages appear to be the drivers of intestinal inflammation in IBD. Macrophages both secrete large amounts of pro-inflammatory factors, such as IL-6, TNF, and IL-1β, and elicit abnormal mucosal responses to intestinal flora (91). The above studies again demonstrate the importance of immune dysregulation and inflammatory response in SLE and IBD.

Moreover, as most of the core genes are low-expressed in the disease group (SLE or IBD), it also means that there will be more neutrophils enriched in the disease. Li et al. uncovered a novel mechanism implicating the dysregulation of neutrophil ferroptosis in the initiation and progression of systemic lupus erythematosus (SLE), suggesting a pivotal role of innate immune cell abnormalities in the disease (92). In parallel, a study by Knight et al. in the context of neutrophil extracellular traps (NETs) highlighted their potential as self-antigens that could mediate organ damage in autoimmune diseases, thus providing a link between NETs and the pathogenesis of conditions like SLE (93). This aligns with the observations made by Pruchniak et al., who explored NET generation and degradation in patients with granulomatosis with polyangiitis and SLE, further elucidating the complex role of NETs in autoimmune diseases (93). Adding to this, research conducted by Gottlieb et al. on neutrophil extracellular traps in pediatric inflammatory bowel disease (IBD) underscores the significance of NETs in the inflammatory processes underlying IBD, pointing towards their potential as therapeutic targets (94). These studies underscore the multifaceted role of neutrophils and NETs in autoimmune and inflammatory diseases, highlighting their potential as biomarkers for disease activity and as targets for therapeutic intervention.

Finally, we downloaded single-cell datasets from SLE patients and performed single-cell annotation analysis to detect cellular heterogeneity and elucidate its underlying mechanisms. Four cell populations including B cells, T cells, NK cells and monocytes, were mainly identified, in which five hub genes were expressed on immune cells to varying degrees, and the expression trends were consistent with the previous analysis. This provides a direction for further study on the mechanism behind the co-morbidity of SLE and IBD.

In summary, this study explored and identified the hub genes of IBD and SLE for the first time, and analyzed the possible pathogenesis. Five key genes, KLRB1, KLRF1, GZMK, IL-7R and CD40LG, may become potential biomarkers. However, this paper had some limitations. Since the vast majority of SLE and IBD patients in the clinic have been treated with hormones and immunosuppressants for many years, this poses difficulties in the validation of the key pivotal genes. In the future, we will collect blood and tissue samples of patients with initial onset of disease to verify the expression and potential function of the hub genes.

## 5 Conclusion

In this study, we found that positive regulation of programmed cell death and inflammatory response may be the common pathogenic mechanisms of SLE and IBD. Also, we established five key genes (KLRB1, KLRF1, GZMK, IL7R and CD40LG) as characteristic diagnostic markers. In addition, SLE and IBD exhibit comparable patterns of immune cell infiltration, which provides direction for future treatment.

## Data availability statement

The datasets presented in this study can be found in online repositories. The names of the repository/repositories and accession number(s) can be found in the article/**Supplementary Material**.

## Author contributions

H-WS: Conceptualization, Data curation, Methodology, Software, Supervision, Validation, Writing – original draft, Writing – review & editing. XZ: Conceptualization, Data curation, Formal analysis, Methodology, Software, Supervision, Validation, Writing – original draft, Writing – review & editing. C-CS: Funding acquisition, Resources, Software, Supervision, Writing – review & editing.

## Supplementary material

Supplementary Figure S1 Veen plots of crossover genes for the four cohort sets.

Supplementary Material 1 .

Supplementary Material 2 A list of DEGs.
