# Supplementary figures and images for: The shared circulating diagnostic biomarkers and molecular mechanisms of systemic lupus erythematosus and inflammatory bowel disease
Source: Front Immunol. 2024 May 7;15:1354348. doi: 10.3389/fimmu.2024.1354348 (PMC11106441; doi:10.3389/fimmu.2024.1354348)

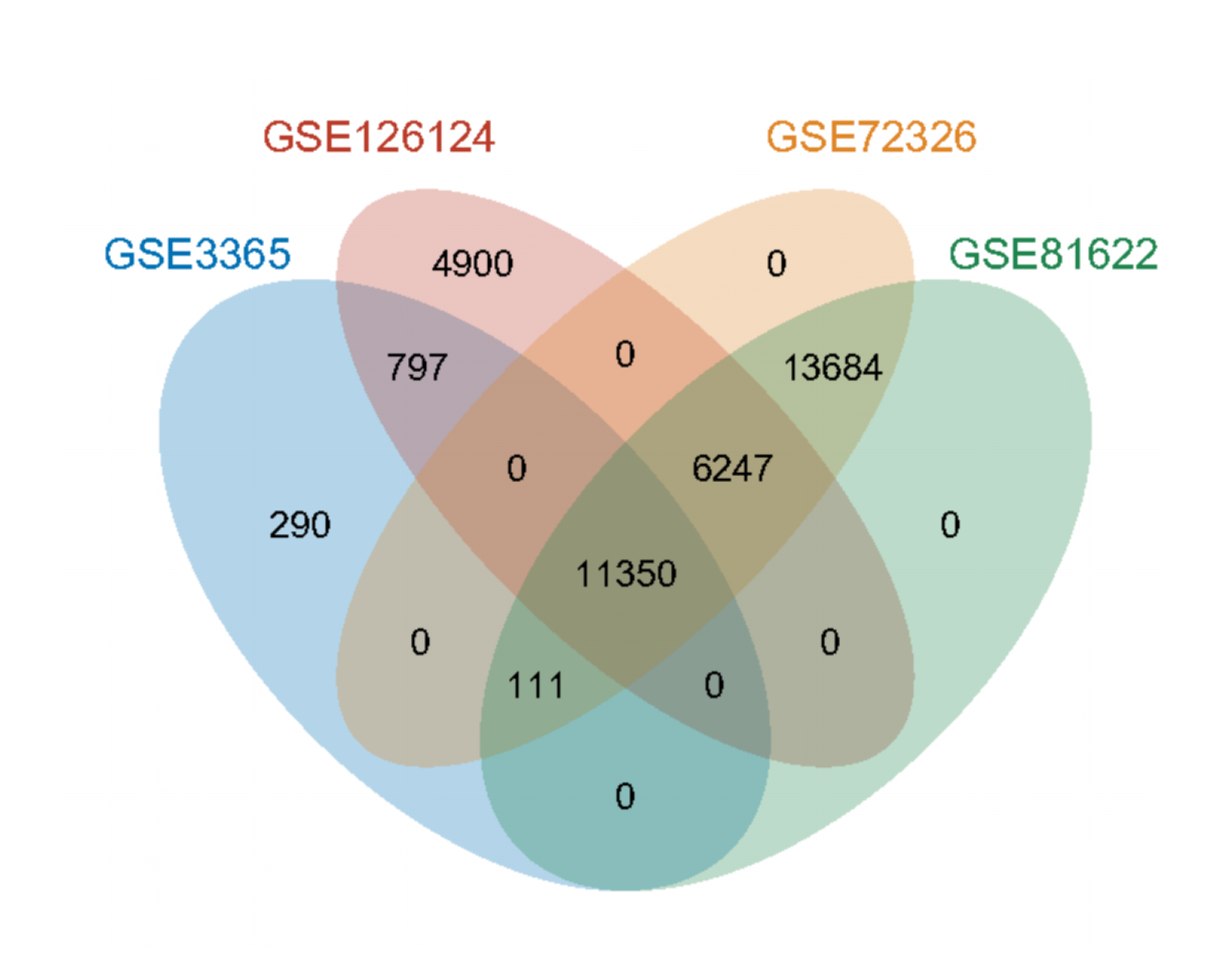

Supplement: Supplementary Figure S1 — Veen plots of crossover genes for the four cohort sets. [file Image_1.png]
